# A Practice of Physic, Comprising Most of the Diseases Not Treated of in “Diseases of Females,” and “Diseases of Children”

**Published:** 1830

**Authors:** 


### REVIEWS AND BIBLIOGRAPHICAL NOTICES.

---

Art. VII. *A Practice of Physic, comprising most of the Diseases not treated of in "Diseases of Females," and "Diseases of Children."* By William P. Dewees, M. D. Adjunct Professor of Midwifery in the University of Pennsylvania: In two volumes. Philadelphia, Carey & Lea, 1830. p. 600.

"We live in an age in which the fear of *debility*, causes a prodigal use of *stimulants*; and this too often, at the expense of health, and the life of the patient."—*Broussais, Phleg. Chron. vol. ii. p.* 82.

"Had I *dared* to bleed freely, and especially by means of leeches, the patient might have been saved; but I was afraid of *debility*. But, who is to blame!"—*Ib. p.* 178.

The Profession have so long been looking for some general work on the Practice of Medicine from the Philadelphia School, that we at length hail with pleasure, the work of Dr. Dewees. His accuracy of observation, his judgment, his lucid descriptions, are surpassed by very few. His copious manner, which pursues a subject throughout all its ramifications, and leaves nothing to be supplied by the judgment or imagination of his reader, may, indeed, be considered as an objection by many; but upon this point *we* are not disposed to cavil with him. The talent for writing rules of practice, concise and comprehensive, is given to few, and the great majority of readers are better pleased, and more instructed by copious illustrations and details.

Dr. Dewees has not pretended to give a very perfect arrangement of diseases, nor a complete view of the state of medicine at the present day. 1. He has omitted the diseases of which he has already treated in his former works. 2. With still more propriety, we think, he has omitted those diseases which have not fallen under his own observation. Every manual of medicine is, at present, burdened with unsatisfactory accounts of the symptoms and treatment of diseaes, which the authors have never seen ; and of which they cannot consequently be expected to offer any thing, original or interesting. The shades of disease are marked by tints of too delicate a shade, to admit of their being faithfully described, by persons who know them only through the reports of others ; and such is the abundance of writers in every department of medicine, that every branch of the profession may be safely left to persons acquainted with the subject from personal observation. 3. Dr. Dewees has very wisely omitted such diseases as his own experience has taught him no successful mode of managing. Of this kind are jaundice, hydrophobia, and almost all the neuralgiæ, &c. together with some others, the treatment of which is not yet settled. After making these deductions, he has selected but about thirty-five out of the numerous diseases which flesh is heir to, as the subject of his work. As the work is designed to be of a popular character, principally for the use of students, we are disposed to consider this as a very decided improvement. These few diseases comprehend, in their treatment, almost all the principles of the science. It is of great importance to the student, on commencing the study of his profession, to have his attention confined to a few diseases, in which he may have distinctly placed before him, the prominent principles of the profession, that he may afterward apply to other forms of disease, when a more improved judgment has taught him to distinguish what is of practical utility, from that which is interesting merely, as matter of general professional information.

But the narrow limits assigned us, require us to proceed to the examination of the work. More than fifty pages of the work are devoted to "General Observations," comprehending every thing that can conduce to the benefit or comfort of the patient, even to the minutest regulation that can affect his convenience—a part of professional duty that is frequently left to the discretion of attendants, to the great detriment of the patient, and the serious injury of the reputation of the physician. Nothing tends so strongly to endear the physician to his patient, and, by securing his confidence to command the regular administration of remedies, as an assiduous attention to those minute circumstances, and a careful anticipation of those little sources of discomfort, which are too often looked upon as beneath the dignity of professional science. He who would be a successful practitioner of medicine, must add to the most extended general views of his profession, the constant habit of minute observation. Sickness always has a tendency to bring the firmest mind down to the feelings and sentiments of childhood; and hence physicians of the slighest qualifications, with nothing to command either respect or admiration, frequently not only secure the confidence of the public, but command a degree of success that many of their superiors might be proud to equal. But our limits permit us to make no extracts from this part of the work. We shall therefore take leave of it, by recommending it to the diligent perusal of the young practitioner, with an assurance that his time could not be more profitably employed.

So far as the theory of fever is concerned, he gives us to understand that he is an unqualified Broussaian; considering it "as settled," "that in all the supposed varieties of fever," the lining membrane of the stomach is constantly found, after death, in a state of inflammation, and that all the phenomena of fever depend upon the altered condition of this organ, and consequently that all the remedial measures are such, and such only, as are calculated to diminish or remove it. And farther, " that every thing which has not this tendency is not only useless, but is injurious."

Practically, however, the author is not so far a Broussaian as to prevent him from attempting to supplant the pathological state, by the liberal administration of purgatives, and occasionally of emetics.

Chapter I. treats of the remedies for fever in general. The following remarks appear to us to be judicious:

"Fevers of every denomination, be their types what they may, frequently have sickness of stomach as an attendant. This nausea, or it may amount occasionally to vomiting, is always attributed by the attendants upon the sick, to "*a foul stomach*," and in their opinions, decidedly calls for an emetic. This, nine times out of ten, is an error; for this sickness, &c., only points out a state of irritation of this organ; and so far from its being relieved by an emetic, is almost certainly aggravated by it. In such cases, cathartic medicines of a moderately active kind should first be given; and if these do not afford relief, try, 2d, the direct application of such remedies as are known for their efficacy in such cases; and 3d, if these fail, counter-irritants must be used." p. 73.

For the first indication he prescribes grain doses of calomel, repeated every hour until eight grains are taken, to be followed, if necessary, by a few teaspoonsful of magnesia and enemata. For the second, small quantities of cold gum arabic water, milk and water, or what is almost certainly successful, the carbonate of soda in combination with gum arab., ol. minthæ, and seltzer or common water, to be taken every half hour or hour, as the symptoms may be more or less urgent. For the third indication he recommends cupping or leeching, sinapisms, and if the sickness be obstinate, a blister.

While on the subject of emetics, we will express the opinion, that besides inflammatory irritation, irritability of the stomach may arise from a want of tone, the consequence of sympathy with that state of the surface produced by the chill. In both these conditions, although emetics are certainly hazardous, yet their great efficacy in intermittent fever, may render us anxious to avail ourselves of their use.

Under the first condition, if the irritation be moderate, the emetic may be rendered safe by general or local bleeding. And under the second, by the liberal use of the vegetable diaphoretics, aromatics, opiates, or camphor. If their use be at all doubtful, antimony should be avoided, or at least be combined with ipecacuanha.

For bleeding in fever, Dr. D. may be considered as a very general, almost a universal, and we think, a very judicious advocate, and his directions in different parts of the work are very explicit. The quantity taken is not to be determined by oz.

but by the effect produced upon the system. In certain epidemics, as well as in certain constitutions, bleeding is, by many physicians, proscribed entirely, because it cannot be used with as much freedom as we are in the habit of employing it upon other occasions. Now we can see no reason why this, like other remedies, should not be accommodated to the state of the system; or why a patient may not derive benefit from the loss of six or eight oz. of blood, who would sink under the loss of twenty oz. The reasoning of Rush, on this subject, if not strictly true to the extent he carries it, at least merits attention; that if bleeding in old and debilitated habits, demands more caution, it is more imperiously called for by the fact that their frail and debilitated organs more readily give way to the violence of inflammatory action. We feel convinced, that from the prevalence of a prejudice of this kind, much suffering has been permitted to go unrelieved, which might have been avoided by timely and judicious depletion.

Under the head of Sweating, Dr. D. combats the very prevalent idea, that the efficacy of the process is in proportion to the quantity of fluid evacuated by the skin.

* * * "In a number of instances we have known an overwhelming sweat to be followed by a hard, rigid skin, and without the smallest diminution of the force of the arterial system, or the least abatement of the distressing and threatening symptoms. On the other hand, we have seen the whole system tranquillized and relieved by a gentle moisture breaking out spontaneously, or when produced by the exhibition of some proper remedy for this end. We may therefore lay it down as an invariable rule, as far as our experience justifies us in the assertion, that in no instance of fever, simply so called, does profuse sweating relieve as much as gentle perspiration. It follows, then, that this evacuation has been in too many instances indiscreetly urged.

In employing remedies with a view to promote perspiration, (for this is all that should be attempted,) we must regard with attentive care, 1st, the period; 2d, the state of the body as regards temperature; and 3d, the agents themselves.

The period of the febrile paroxysm at which we attempt its solution by this remedy, is a matter of great moment; we must, therefore, not commence our operation in the cold stage; nor should we be more successful at the formation of the hot stage, nor at its height, unless proper evacuations have so much reduced the vigour of the pulse, as to render the operation of the medicine probable; or unless the fever itself is of so mild a grade, that an impression can be made upon it by the exhibition of diaphoretics. It therefore follows, that we should not exhibit any one of this class of medicines while the pulse is full, hard, and frequent—for were we to do so, we should not only be foiled in our attempts, but have the mortification to see every symptom aggravated; for there is a "sweating point" of the arterial system, as well as a "sweating point" of temperature." p. 77—78.

It is very necessary to attend both to the period of the paroxysm, and the period of the disease; yet his prohibition of diaphoretics in the cold stage, is certainly too general. Diaphoretics, in the practice of every practitioner much conversant with intermittent fever, have proved successful in breaking up the disease.

The temperature of the body favourable to perspiration, he thinks is fixed too high by Dr. Alexander, at 108, and that 100 or 102, is nearer the truth; and hence when the temperature is above this point, diaphoresis will best be promoted by reducing the temperature.

Among the agents for producing perspiration, he mentions opium in the following language:

"If there be but a moderate degree of vigour in the pulse, or if it be soft, opium is a highly important drug. This medicine seems to merit a decided preference in all the more protracted forms of fever, or where the powers of the system have been pretty much expended; and in all those of weak action, even in the commencement. The proper time for its exhibition is at the early part of the hot stage. It should be given in such quantities, and at such intervals, as to make a decided impression; but not so much as to have its narcotic effects predominate; nor should the frequency of exhibition be such as to subject the system to this part of its influence. We therefore hold it wrong to give it, either in such a dose or doses, as will subject the brain to its anodyne powers; for whenever we induce this tendency to coma, we unnecessarily prostrate the system." p. 79—80. * * *

We shall permit him, however, to specify its application. Under the head of Intermittent Fever, after alluding to the practice of Dr. Lind, of giving opium during the hot stage, he remarks:

* * * "In this particular district of country we are of opinion, it should be given with caution, where bleeding, purging, and other depleting remedies, have not preceded its use. We have seen the most decided good effects of this remedy after a sufficient bleeding, and would never hesitate to exhibit it where this evacuation had diminished the force of the pulse; and where there was no marked determination to the head. In the warmer parts of our country, we need not perhaps be so cautious—as there, the disposition to inflammatory diathesis is less; and opium can be given both earlier and with greater freedom. Besides, they resemble more the climates in which Dr. Lind found opium so useful.

If, then, the pulse be moderately soft, either from the disease not being much disposed to inflammation, or from previous depletion, a grain of opium, with a quarter of a grain of the tartrate of antimony, may be given at the commencement of a hot fit; and if it act favourably, it will procure a free perspiration over the whole body—but if instead of this, we find the hot stage protracted, or all the symptoms aggravated, it should not be again given, until the activity of the blood-vessels is more effectually subdued.

It may not be amiss to say, that the epidemic character of intermittents, should always be consulted when we propose to use opium in any shape, for it is one of those remedies which has a very decided agency upon the nervous system; and when exhibited in any acute disease, without immediate benefit, it rarely fails to do harm—therefore, the influence of the epidemic upon the force and vigour of the pulse, must always be taken into consideration. And as a general rule, we may with safety declare, that the usefulness of opium in arresting the paroxysms of intermittents, will be almost in proportion to the reduction of the pulse." p. 91.

And again, after speaking of the importunity of the patient for opiates, to allay the restlessness and inquietude of the sleepless nights in remittent fever, he remarks:

* * * "An ill-timed exhibition of opium in certain states of the remittent form of fever, is almost always mischievous; and we are sorry to add, has been too often fatal. We have seen, without the smallest doubt, several instances of heavy stupor, and apoplexy, produced by even a dose of laudanum.

On this account we are extremely reluctant to give it, at almost any period of a remittent; and more especially to a yellow fever patient, whatever may be the degree of sleeplessness or agitation. Under such circumstances, we have found, almost constantly, that there was either a general or partial accumulation of heat, especially of the head, a dry skin, bowels rather tardy, or very offensive evacuations, to be the cause. Or it has been found owing to a dull but pretty constant pain in the head, attended sometimes by slight delirium; and other times with a slight stupor, alternating with distressing watchfulness. Pulse either too quick or lagging.

In these cases, we are certain that opium would be injurious, if not fatal. Instead, therefore, of it, in such cases, cold applications to the heated parts of the body, and extremities, by sponging; to the head by wetted cloths, or a bladder of cold water; a few leeches should be applied to the temples; a mild purge should be exhibited; as castor oil; a free ventillation of the room should be made, together with a decided reduction of the bed-clothes." * * *

In intermittent fever, we think that the author lays too much stress upon the pulse, as a guide to the use of this medicine, which, in fevers of this description, is a very uncertain criterion to judge by, and that he attributes too much to the influence of climate in modifying autumnal disease; the physicians in the latitude of S. Carolina and Georgia, being as much afraid of the use of opium, as in Philadelphia.

On the subject of purgatives, he is very explicit as to their importance, but he is rather indefinite as to the kind we should use. Entertaining a very great dread of drastic purgatives, he recommends moderate purging by grain doses of calomel, repeated hourly until six or eight grs. are taken, to be followed by castor oil, epsom salts, and magnesia, or some other mild cathartic. We must consider the division of this class of medicines into laxatives and purgatives, as a very superficial one. If they differed only in degree, the former, by increasing the dose, might be made to answer all the indications of the latter. With some of these, this would be actually the case, if their inefficient nature did not, under certain circumstances, preclude the possibility of taking them in sufficient quantity. With others, however, as with the neutral salts, the serous exhalation they produce is entirely out of proportion with their effect in evacuating the bowels, and hence patients are flattered with the appearance of copious evacuations, while the end for which they were prescribed is entirely unanswered. By an active course of general and local bleeding, by the use of antimonials, and the most rigid abstinence, the more drastic cathartics may perhaps be dispensed with; but their use should be relinquished only by those who are disposed to follow out the Broussaian practice in all its details.

On the subject of arsenic, Dr. D. remarks:

"As far as our experience enables us to institute a comparison between it and the bark, we should say, it is fully as certain, and without some of its inconveniences. The only objection that we believe can be urged against it, is, that it is a medicine of most deadly power when improperly used. But when properly exhibited, it is as safe as opium, or any other medicine in daily use. We have never seen the least injury result from it—it sometimes sickens the stomach, and occasionally even to vomiting—but of what medicine of any power may not the same be said.*

The arsenical solution has a decided advantage over any preparation of bark, when children are the subjects for its exhibition—to them it can be administered with the fullest effect, without their being aware they have taken any thing. It may be given when the system cannot receive the bark, or even when the pulse is too full to bear its use."† p. 96.

Our limited experience with the article would lead us to prescribe it, precisely as we would give digitalis in dropsy; when the symptoms of inflammation were subdued, and the disease had assumed a chronic form.

On the subject of relapse in intermittent fever, he thinks they are particularly apt to occur on the 7, 9, and 13 days, and recommends these days to be anticipated, by a few doses of the bark; by the black pepper, of which he recommends 6 or 8 grains to be taken three times a day; or the piperine in doses of 1 grain in the same manner.

Chapter IV. *On Yellow Fever.* Our limits permit us only to glance at the contents of this chapter. The author adopts the idea that this disease and the bilious remittent, are distinct diseases; admitting at the same time that the distinction is of little practical importance, since the treatment is to be conducted on very nearly the same principles. The following extract will give some idea of his classification of the varieties of the disease:

---

"*When we have witnessed the most decided good effects from this medicine, it has always been in recent, or at least, not in very long standing cases—and we think it more successful in young, than in old subjects.

†The arsenic, like every other tonic or stimulant, employed for the cure of fever, must have the system prepared for its reception; but we think it can be given earlier, and with less depletion, than the bark. Nor is it a matter of any consequence, so far as we have observed, if the fever be not absolutely subdued at the time of its commencement. Indeed, some are of opinion, that this medicine can be given during the whole march of the disease. As regards our own experience, we confess it to be at variance with this declaration."

Three distinct modes of attack may be observed in the yellow fever, as we have just observed ; each of which has something peculiar to itself ; this variety must arise from, 1. The greater or less degree of concentration of the remote cause, or marsh miasma. 2. To the peculiarity of constitution. 3. Or to the nature and degree of the exciting cause. Each of these circumstances will necessarily modify to a certain extent, the form or force of the disease ; accordingly we find it presenting itself, 1. Where the disease rapidly runs on to dissolution, and is accompanied by black vomit—this form has often terminated its career within three days, and never exceeding the fifth day. 2. Where the disease was without remissions, or when they were so indistinct, as scarcely to be observed. In this form of the disease, the course is not run with so much rapidity as the first ; but for the most part the sufferings of the patient are greater—there is no perceptible attempt at crisis, and where it terminates fatally, it is for the most part from the fifth to the seventh day, and is not necessarily attended with black vomit. 3. Where the paroxysms can be pretty regularly traced, or where the periods of exacerbation are not so entirely uncertain, but where there is stronger evidences of an inflammatory diathesis, than in either of the former ; but which may rapidly change if not arrested by proper remedies, into an opposite state, and terminates either in the black, or the coffee-ground vomit—this form may terminate within five days, or if checked, may run on to the seventh, ninth, or eleventh day." p. 126—127.

As to the treatment, Dr. D. recommends a decided antiphlogistic course, bleeding in the cautious but decided manner, recommended by Rush ; purging with calomel, followed, if necessary, by other mild purgatives, &c.

The plan which the author has pursued in this work, of confining himself, except in a few instances, to the record of his own experience, has prevented him from frequent allusions to the opinions and practice of others. We must confess, however, that we felt disappointed, that in treating of this disease, he has made no allusion to the character of Dr. Rush. The principles for which this illustrious man contended, are now so universally admitted, that few are sensible of the extent of our obligations to his memory, and in no part of the United States is the merit of this physician more overlooked, than in the city of Philadelphia. Some tribute of respect, therefore, under these circumstances, was the least that might have been expected from one who bore with Dr. Rush the brunt of popular prejudice in '93, and who is himself so honorably mentioned by him in his account of that and the succeeding epidemics.

Chapter II, on *Continued Fever*. Dr. D., following the authority of other writers, divides this form of fever into the *Inflammatory*, *Synochus*, and *Typhus*. For the cure of the former he recommends bleeding, *to an extent that will insure an abatement of the headache, and decided reduction of the pulse*; local depletion by cups and leeches; free purging by calomel and *the more mild purgatives*. Calomel is to be dispensed with after the first or second day, *unless the evacuations become dark coloured, without odour, or very offensive*, with a *frequent inclination to use the pan without passing much at a time*. Under these circumstances, it should be given, and continued until it produces a change in these respects. He advises emetics under the following circumstances.

"Should the bowels not be speedily or sufficiently obedient to the medicines exhibited, but become painful and tumid, they should be excited to discharge themselves by means of a simple injection. If the stomach become very sick, and throw up bile, twenty grains of ipecacuanha should be given in a table-spoonful of lukewarm water, and its operation encouraged by draughts of warm water. But if the stomach be merely sick, or rejects a colourless or pea-green fluid, the emetic should not be given; especially, if there be a tendency to dryness of the tongue, or much tenderness at the pit of the stomach." p. 148.

Dr. D. admits the *typhus* form of continued fever into this division, with great reluctance, as he believes it to be, not the necessary consequence of any particular form of fever, but wholly the effect of injudicious or inefficient treatment. We cannot agree with Dr. D. and others who refer this form of fever to a state of previous excitement; for at times, when inflammatory remittent fever has been epidemic, we have seen numerous cases among persons who have been exposed to long continued cold, or to cold alternated with heat, that have assumed this typhus form, most unequivocally, from the commencement.

How are we to reconcile the following qualified remarks, with his unhesitating belief in the gastric origin of fever, as expressed in a former quotation.

"The disease in question, has an anatomical character without doubt; and though, we do not consent either to Broussais, or Clutterbuck's exclusive locations, for this character, (as both are certainly right at times,) yet we are persuaded, that in every instance of this disease, some one portion of the system, has been acted upon in an especial manner by the remote cause; which will have the effect perh ps, of modifying the force, and perhaps the succession of phenomena attendant upon the disease when about to be developed, or after this has fully taken place." p. 157.

We shall extract from this chapter only the following summary of general principles, to which, with some slight qualifications, we are disposed to give our cordial assent:

"First. That in all adynamic fevers, there is more or less inflammation almost constantly present, either general, or local.

Second. That even during this more or less inflammatory condition of the system in general, or in portions of it, that the most unequivocal evidence exists, that, a certain combination of symptoms will present themselves; such as a dry parched tongue, and lips; flushed cheeks; low, muttering delirium; a dry, rough, unrelenting skin; lying upon the back, with the legs drawn up; high colored, scanty urine; subsultus tendinum, picking of the bed clothes; and which symptoms have constantly, or with very few exceptions, been called typhus, or typhoid symptoms.

Third. That the symptoms just enumerated, do not give evidence, that all inflammation is at an end, when they make their appearance, as is generally supposed.

Fourth. And that when the treatment is made to confirm to such opinion, by the employment of tonics, and stimulants, that the most serious consequences have followed the practice.

Fifth. That though the symptoms mark a less active state of inflammatory action, yet that the action present, nevertheless, is of the phlogistic kind; and cannot be relieved by a stimulating plan of treatment.

Sixth. That nothing in practice is more decidedly wrong, or more actively mischievous, than the belief, that, when the system will not bear active depletion, that it necessarily calls for the opposite mode of treatment.

Seventh. That it is now agreeable to the best experience, that the employment of stimulants, is sure to be followed by a marked increase of every bad symptom; and that when recoveries have taken place under the stimulating plan of cure, they have only marked the strength of the recuperative powers of the system, and not the eligibility of the plan adopted.

Eighth. That when the system is in that forlorn condition, in which we dare not deplete; and that we must not stimulate, it is the best and most successful practice, to remove all the physical causes in our power, that may have a tendency to depress the oppressed system; such as impure air; offensive smells; soiled clothes; too much heat; too low a temperature; too great a weight of bed clothes, and too much company. To administer such kind, and quantity of nourishment, from time to time, as will offer the least trouble to the feeble digestive powers; (such as has already been specified at par. 217,) and such drinks, as will make an agreeable impression upon the gustatory nerves; but which shall not convey to the stomach any decided stimulating agency. To promote to the last, the alvine discharges, either by injections, or by the mildest aperients; to effect, (and if necessary by even artificial means,) the flow of urine." p. 165—166.

Chapter VI. *Typhus.* Under this head, the author has deviated from the principle laid down in his preface, by treating of a disease which he believes to be peculiar to Great Britain and to Denmark, and which only makes its appearance in other countries when introduced from one of these.

His description of the disease, and the mode of treatment, is principally drawn from Dr. Burne's Treatise on Adynamic Fevers. We shall, therefore, pass it by without comment, except to remark, that in the conciliatory paragraph, he has certainly done the physicians of this country great injustice.

" 'We have thus given the general outline of Dr, Burne's account and treatment of typhus fever; it will be quickly perceived, that his plan is very different from that, which is generally pursued in this country by many practitioners for this supposed disease as supervening upon our Mismatic fevers, and as we have had occasion to remark at p. 152 et seq. With those who dread the onset of typhus in every species of fever; this practice will not be altogether approved of—" what," say they, " bleed, cup, and purge, when typhus is impending! No, no, give bark, wine, volatile alkali, brandy, ether, phosphorus, &c. &c. if you mean to cure the disease.' "

We do not believe that a physician of any intelligence, or character, could be found to use this language. in the United States.

Chapter VII. *Measles.* This, Dr. D. states, generally makes its appearance in the winter, and is more severe in cold weather than in mild. There is a popular opinion, for which he thinks there is some foundation, that it returns regularly as an epidemic, once in seven years. Upon the authority of some experiments of Dr. Chapman, in which the blood, the tears, the mucus of the nostrils and bronchiæ, the eruptive matter of the cuticle properly moistened, were all tried, and without success, he is disposed to doubt its advantageous nature.

As regards the prognosis, he observes, that the disease is dangerous in proportion as the head, the lungs, and the stomach, may be affected, and is always to be feared in habits disposed to consumption. Much fever, without a corresponding eruption, or if the eruption come out with difficulty, or of a pale or livid color, is always a bad sign. The same may be remarked of the abrupt disappearance of the eruption, or if it be attended with severe vomiting, tenderness of the epigastrium, or a diarrhea.

The following may be considered as a very favorable specimen of the Broussaian mode of reasoning:

"Death, when it happens, however, takes place in measles at different periods in the progress of the disease, which produces a difference in the phenomena observable in examinations; thus it has been known to prove fatal as early as the fourth day. When this event takes place so early, it is generally attributed to the "striking in" of the eruption; but this is not the case, were we to adopt even the popular language upon this subject; for when a disease leaves the skin, or more properly speaking, when it is no longer maintained there, it is not because it has changed its seat, but because some portion of the mucous membrane may be too powerfully affected by an efflorescence similar in its nature to that which occupied the skin.

In this case the peculiar action, or inflammation constituting measles, so far transcends its ordinary degree, as to destroy its peculiarity of action in either the mucous membrane of the bronchia, or of the stomach, or intestines; and consequently the peculiar sympathy of the skin which gave rise to the eruption upon its surface, could no longer be maintained, because the specific action on other parts, and from which the sympathy arose, no longer existed, in consequence of the inordinate or altered action in the original seat of irritation. The same thing occurs in variola." p. 183—184.

Dr. D. lays it down as a general rule, liable to exceptions imposed, by the constitution, season of the year, and above all, by the epidemic peculiarity of the atmosphere, that measles is more inflammatory than any other of the eruptive diseases. Hence when symptoms of local inflammation appear with violence, the antiphlogistic mode of treatment is to be enforced in its utmost rigor, and it frequently becomes necessary to do this, when the system is so much oppressed as to call, apparently, for treatment of a very opposite nature.

The character of the disease may be typhoid; when a free use of the lancet would be improper. We must then have recourse to cupping, and blisters, if there be much pain or oppression about the chest, emetics if there be great accumulation of phlegm, plentiful purging with calomel, and the warm bath. Should symptoms of exhaustion supervene, diffusible stimuli may be used, with blisters to the extremities, or sinapisms to the soles of the feet.

As soon as the inflammatory symptoms are subdued, opiates may be resorted to, in that form which is least objectionable to the patient.

Cough, or diarrhea, are very liable to follow this disease, and as they frequently run to a fatal termination in a very insidious manner, they should be met, when at all violent, by very prompt and decided measures.

The patient, in this disease, should be kept cool, but the tendency to affections of the lungs, forbids that he should be kept so as much as in small pox. From sixty to sixty-four degrees of Fahrenheit, is the proper standard.

Chapter VIII. *Apoplexy.* In this chapter, Dr. D. having detailed the history and predisposing causes of apoplexy, gives, at some length, the opinions of M. Serres in regard to its pathology. This writer supposes that effusions are the effect, and not the cause of apoplexies. In support of these opinions, he alleges experiments made upon animals, by causing effusions from the longitudinal sinus, artificial effusions in the ventricles, and artificial excavations in the cerebral substance,—in which no apoplexy was produced. He refers also to the numerous cases to be met with in the annals of our sciences, in which very considerable effusion has taken place without apoplexy, and again of apoplexy taking place without effusion. He divides apoplexy into two species. 1. *Meningeal Apoplexy*, in which the membranes are diseased. 2. *Cerebral Apoplexy*, in which the disease is located in the membranes, and makes the following distinction between the two varieties:

"'The meningeal apoplexy principally attacks youths from the age of fifteen, and old men past sixty—it generally affects females before the last named period." "Of forty meningeal apoplexies, there were thirty two in females, and eight in males.'

*Attack*.—"Physicians have differed greatly in the mode of attack in apoplexies. One party has said that they always come on suddenly—the other, that the attack is preceded by precursory symptoms, which manifest themselves many days previous. Both sides have been right, and both in a certain degree wrong, as the cases may have been meningeal or cerebral."

The meningeal apoplexy is almost always slow; has precursory symptoms, as torpor; difficulty in exercising the mind, and its becoming easily fatigued; perception blunted; drowsiness; respiration slower than ordinary—tardy circulatlon; less warmth than usual; secretions diminished; impaired digestion, and sometimes vomiting.

Mr. Serres asks, "what distinguishes apoplexy from sleep?" He answers the question by the following important observations. "In sleep the respiration is slow, and the circulation is in a relative proportion—in apoplexy the natural relation is destroyed."

Whatever difference age and strength may make, in the number of pulses in a given time, the discordance between them and respiration never fails to show itself; and when this is at its maximum, the stupor is also. Thus Mr. S. relates a case in which the following extreme of disparity between the pulse and respiration existed—pulse, eighty-three strokes to eleven inspirations. And that the abolition of the natural and mental functions, are in the precise ratio to the loss of the healthy proportions between the motions of the heart and the lungs; and that these functions are restored, exactly in the degree that the pulse and the breathing approach the natural standard.

He also states this curious, and to diagnosis, valuable fact: that in meningeal apoplexy, respiration is always equal on both sides; that is, the thorax is equally dilated on the right and left sides, which is not the case in cerebral apoplexies. The mouth is not distorted; the body lies in a straight line; and if the patient be not in a state of stupor, he will present both hands—if he be somnolent, irritating the limbs will produce the same motions. The nervous and muscular systems preserve their powers on both sides. Though meningeal apoplexies present important varieties in the fluids effused, Mr. S. acknowledges he has never been able to indicate them individually from the accompanying symptoms.

The varieties of meningeal apoplexies, are deduced from the nature of the fluid effused; by the absence of effusion, and the rupture of arteries or veins in the brain. Hence there is, 1st, *meningeal apoplexy*, without effusion; 2d, with effusion of simple serosity; 3d, with sero-sanguineous effusion; 4th, with arterial rupture, or aneurismal dilation; 5th, with venous rupture." p. 205—206.

*Cerebral Apoplexies and their varieties.*—" The attack is often instantaneous, especially in men of plethoric habits, short necks, corpulent, and addicted to wine and women. Some moments before the attack, the mind is more than usually active. Sometimes a numbness of one side, one side of the face, or a fixed pain in the head, precedes the attack; the tongue is sometimes embarrassed; a difficulty in pronouncing certain letters, or words—rarely stuttering.

" But whether the fit has been preceded by these symptoms or not, at the moment of attack the face is coloured in an unusual manner; the cervical and facial veins swell; the tongue becomes embarrassed; the sight is imperfect; the hearing impaired; the sensibility and the faculties of the mind lost—and if the patient be erect, *he falls upon the side which afterwards will be struck with apoplexy*—this circumstance is of much importance to the physician when called to the patient."

" Some hours after the attack, if the brain has not already been destroyed at some part of its surface, respiration becomes slower, the venous blood suffers a mechanical obstruction, and requires an appropriate reaction of the breast. The pulse is strong, hard, and frequent—the action of the heart increases in proportion to the difficulty of respiration. The force and hardness of the pulse continue until the moment a vessel gives way in the brain—it then becomes suddenly small, concentrated, and frequent."

" Respiration is equal on both sides in the beginning of the attack, but the thorax and lungs are unequally dilated—one side of the chest becomes motionless, while the other seems to redouble its activity; on the side in which the action is diminished, the ribs are flattened, but on the opposite side they are elevated; the two sides thus offering a very obvious contrast. This happens previously to the occurrence of the hemiplegia; and it is important to attend to this symptom, as it points out the side that will be paralyzed."

Coma and stupor extreme—sensibility diminished on both sides; sometimes remarkably so on the side about to be injured—at other times sensibility remains, though paralysis is about to take place. The paralyzed member sometimes preserves its sensibility; but when it loses it, it is before the loss of the power of motion." p. 207—208.

In meningeal apoplexy, the membranes of the brain are variously affected. They are thickened, or dry; the vessels are distended; symptoms of inflammation or irritation have taken place; and frequently, effusions of serum, or blood, within the cavities.

When the irritation has been intense, or the membranes inflamed, partially or universally, the effusion is either sanguineous or sero-sanguineous, and is confined to the seat of the irritation.

In the treatment of apoplexy, Dr. D. has suggested one view which we believe will be novel to many of our readers. He states, that a state of depression, similar to that arising from concussion of the brain, often follows the stroke. "The pulse is not in a state of depression, (oppression?) agreeably to his notions of the arterial system." Under these circumstances, he does not venture to draw blood, until by external stimuli applied to the lower extremities, the system is enabled to react.—This distinction we think is not often made. Sir Wilson Philip, in his "Treatise on Indigestion," describes a form of apoplexy as occurring in debilitated habits, with very similar symptoms, and very closely resembling nervous apoplexy. So far however from advising this cautious mode of proceeding, he recommends the distinction between this and nervous apoplexy, to be very carefully made, in order that the lancet, which he looks upon as almost the only resource, may at once be used. Emetics, Dr. D. has never had courage to employ, and thinks that even in *crapulous apoplexy*, in which alone they can be employed with a probability of advantage, they should not be given but after pretty ample depletion.

Chapter IX. *Of Scarlatina.* Dr. D. does not believe this disease to be contagious, "as he has never seen any decided proof that the disease has communicated itself in this way."—For the treatment, he recommends bleeding, if the pulse be full, tense, or hard; emetics; laxatives of which, on account of the tendency to visceral congestion, calomel is most suitable; and cold affusion or sponging if it do not occasion chilliness. The sore throat, as it is not accompanied by pneumonic symptoms, is no objection to this treatment; on the contrary, he has thus seen it most evidently relieved:

"We are told the warm bath is exceedingly efficacious, when the eruption imperfectly takes place, owing to general languor; and especially when attended by coldness of the surface—or having appeared, suddenly recedes, inducing great gastric distress, and other very unpleasant symptoms—to cleanse the foul ulcers of the throat, emetics are found most effectual—the emetic may be followed by the use of detergent gargles; the best of which are composed of Peruvian bark, with a portion of the tincture of myrrh—or barley water, acidulated with the sulphuric or muriatic acid, with the addition of honey. An infusion of Cayenne pepper, alone, or mixed with barley water, or the decoction of bark, is much, and we have reason to believe not too much, praised as a gargle, as far as we can rely on our own observations.

In the malignant shape of this disease, the general practice is nearly the same as in the preceding or anginose state. We rely mainly on evacuations of the primæ viæ—first, by emetics, and next with the mercurial purges."

When great depression supervenes upon either of the forms of the disease, the system must be supported by camphor, ammonia, turpentine, bark, and wine, aided by the ordinary external irritants.

The œdematous swellings of the extremities which frequently follow this disease, are best relieved by laxatives, followed by digitalis.

The dropsical affections frequently arise from the inflammatory stage not having been sufficiently reduced by depleting remedies. When this is the case, they are best relieved by purging, and by diuretics of the "saline kind," as nitre, or nitre and squills.

Chapter X. *Urticaria.* Nettle Rash frequently attacks children who are teething, and who are indulged with much fruit, or acescent food, especially in the summer season. It is frequently occasioned very suddenly by articles of food, by boiled cabbage in the spring of the year, by cold lemonade when the body is heated, and by eating certain kinds of fish. Dr. Hewson mentions several cases of eruptions which arose from taking balsam copaiva, some resembling urticaria, others erythema, or roseola. Strawberries sometimes give rise to it. This disposition to urticaria, generally depends upon idiosyncracy, and is frequently confined to a single article of food. In other individuals there is a general predisposition to the disease, when any thing indigestible has been received into the stomach.

In this disease, there is almost universally acidity of the stomach; hence the general treatment should be alkaline, magnesia, lime-water, and milk; saline purges should be avoided, and in their stead, magnesia, or magnesia and rhubarb, should be used; nothing acid should be taken into the stomach. To relieve the itching, he recommends sprinkling the surface with well toasted rye or wheat flour. We have found nothing so effectual, as gently stimulating the surface with turpentine, or some of the preparations of ammonia. In chronic urticaria, great attention must be paid to the diet, and the condition of the digestive organs. When these measures fail, great benefit has resulted from the persevering use of Fowler's solution.

" A very interesting case of chronic urticaria is related by Cazenave. "In the hospital of St. Louis, in a patient of Mr. Biett's wards," says Mr. C., "we have seen it (urticaria,) accompanying a quotidian intermitting fever, and after having lasted for four years, finally induce swellings and great distention, ecchymoses, ruptures, and ulcerations. In many paroxysms it was accompanied with a general tumefaction; sometimes to such a degree, that the patient was nearly suffocated; his respiration was hurried, the movement of the thorax very slight, the neck swelled, the face puffed up, and of a violet colour, the pulsations of the heart intermitting, and at times scarce perceptible, and death, which appeared imminent, only prevented by large bleedings."

'This patient, who had passed through several hospitals, and in which every means of cure had failed, was at last restored to health by the use of Fowler's solution.' "

Chapter XI. *Phrenitis.* This chapter we are compelled to pass over very slightly, and we do this the more willingly, as the symptoms of the disease are familiar to every physician, and the treatment decided with as much accuracy as the imperfect nature of our profession will probably ever admit of. But although the characters of the disease are generally so distinctly marked, yet occasionally the greatest ambiguity prevails.—*Post mortem* examinations have frequently shewn that the most decided symptoms of inflammation of the brain, have been produced by inflammation of the stomach, intestines, liver, or lungs, while on the other hand, all the consequences of inflammation are frequently met with, when the disease has proceeded so insidiously that its existence was scarcely suspected.

From his treatment we can make but one quotation:

> "Blisters as revulsives are highly useful when the system is reduced to the blistering point. (See p. 260.) They should be applied to the calves of the legs, to the inside of the thighs, or to the forearms. We have strong doubts of the propriety of blistering the head— indeed, we are of opinion that it is injurious; to the shoulders, is less objectionable, though not a decidedly eligible spot." p. 240.

Advice that has been repeated sufficiently often to be familiar to every physician, but which we have too often seen neglected with the most unfortunate consequences.

Chapter XII. *Hydrocephalus Internus.* This chapter we think one of the most valuable in the work. Indeed it was in this chapter that we first recognized the familiar and peculiar manner of the author. The subject, however, has received so much attention from the profession, that we will be justifiable in making but few extracts. Dr. D. never employs bleeding from the arm in this disease, if the system be prostrated before the attack; but bleeds cautiously with leeches, according to the urgency of the symptoms. The following remarks on purging are very judicious:

> "This remedy cannot be dispensed with, in either form of hydrocephalus—in the acute, it is essential, as no other remedy with which we are acquainted, relieves the head of its superfluous blood, like purging—this effect is not difficult to understand if we call to mind the direct communication of the blood-vessels of the abdomen and the head. * * * But notwithstanding our conviction of the efficacy of purging in hydrocephalus, yet it must be understood, that it is only *certainly*, and extensively useful, in the idiopathic acute hydrocephalus, *after the system has been lowered by a previous bleeding, or bleedings.* Not so, perhaps, in the symptomatic species—in this variety, purging may be of paramount benefit to bleeding. * * *
> * * * After a free purging has been instituted, and we think it no longer desirable to persevere in it, we maintain a sufficient action of the bowels by very minute doses of calomel, that is, from a quarter to half a grain every two or three hours, or by small doses of castor oil. It may be proper, however, to suggest a caution to the young practitioner here, not to persist too long in cathartic medicines, by reminding him, that in most instances, the stools will appear of a dark-green colour, purge as we may; therefore, that further purging is not called for from the mere appearance of the evacuations." p. 252—253.

The part we have marked in italics, conveys an important practical precept, from inattention to which, the followers of Broussais having been led into many extravagancies, on which they are constantly harping with much arrogance and little reason. But we shall again refer to this subject at the end of the review.

His remarks on blisters we consider of so much importance, that we shall repeat them, although very similar to those extracted from the former chapter. Indeed we are convinced that there is no remedy from which so much benefit is expected, and so little advantage derived in this disease, as from blistering.

"Blisters are to be applied to the legs and thighs alternately, if necessary, as soon as the first stage threatens a conversion into the second; and they may be repeated to the end of the disease. Many prefer the head; but this is certainly an inconvenient part to blister, if it be not an improper one; and if we can place reliance upon our own experience, we have thought it injurious in many instances. To the nape of the neck, is much better as regards effects; but the position is certainly a fatiguing one to the patient—we have, therefore, for the last thirty years, rarely applied them to this part; believing most firmly that more advantage is derived from their employment upon the extremities." p. 254.

Chapter XIII. *Diseases of the Eyes.* This treatise, which we are given to understand was written by Dr. Hays, we shall pass over, as we intend to take up the subject at large in a future number.

Chapter XVII. *Cynanche Trachealis.* Every physician who has had an opportunity of witnessing the progress of this terrible disease, must be anxious to gain information upon the subject from every source. Dr. D. was led to pay particular attention to it, from having lost one of his own children by it; and we can safely recommend this treatise as a most valuable one. The disease may make its attack in one of two ways.

"This disease attacks in one of two ways; 1st, by a hoarseness, which is perceived upon coughing, and which may continue without increase, for even several days, or until perhaps the sudden application of some exciting cause; such as a change in the temperature of the air.

For exposure to cold and damp, or to check of perspiration, calls forth some of its more formidable symptoms; as more or less difficulty of breathing; an increase of cough without expectoration and fever.

Or secondly, it may attack with the most alarming suddenness where no such onset was expected. When it is thus prompt in its appearance, it menaces life from the moment of its invasion; and if its terrible march be not speedily arrested, it but too frequently triumphs in death." p. 346.

To this hoarseness, as a premonitory symptom, he attaches great importance, and thinks it should never be neglected. We select the following remarks:

" The hoarseness which disappears spontaneously, is very distinct fromthat of croup ; the difference, however, cannot well be conveyed by words, unfortunately, sometimes, for those who may be assailed by it. It may, however, be observed, that there is a certain clearness and distinctness in the croupy sound, that does not attend the other : the one, (the croupy, seems as if it issued from a metallic instrument ; and the other from one of a less vibrating material. The ear, however, by long habit, may learn to distinguish between them, and when once instructed in this discrimination, never loses its tact.

We may also observe that the evanscent hoarseness is almost always accompanied by a little soreness of the throat ; while that of croup, we believe, is never. Again, the first is perceived in common speaking ; whereas that of croup is only discoverable in the commencement by coughing. Lastly, some little pain and soreness are observed about the posterior fauces after coughing, which never happens in that of the croup. It may not however be amiss to observe, that a mere loss of voice must not be mistaken for croupy hoarseness, as we have known it to be on several occasions, to the great terror of an anxious parent." p. 349.

Dr. D. divides the disease into three stages. *The First, or Forming Stage ; The Second, in which the disease is completely formed ;* and the *Third, or Congestive Stage.* Upon what principle this last stage is called *Congestive*, we are at a loss to determine. Dr. D's practice does not differ materially from that generally recommended. When the disease is decidedly formed, he recommends a large bleeding to make the first impression. But he cautions the young pracitioner against making *mere difficulty of breathing* the only indication for a second bleeding.

"To make a second bleeding proper, there must be a continuation of the same symptoms, though perhaps with a lesser degree of force, which made us determine upon this operation in the first instance; that is, the pulse must be firm, the skin warm, the face flushed and the oppression considerable." p. 363.

He is decidedly opposed to leeches, and if topical bleeding is necessary, recommends cupping between the shoulders.

Persons of more experience than ourselves must decide upon the propriety of the following condemnation of the warm bath. But in confirmation of his views we may remark that it has seldom fulfilled our expectations.

"This, of all the remedies employed in croup, requires the most judgement in prescribing it; and certainly the most caution, properly to apply it. We have never seen it managed with so much address, as not to have made us tremble for the consequences; nor with so much success as to tempt us to brave them. We can most conscientiously declare, we have never, in a single instance, witnessed a decided advantage to arise from its application; but we can most truly say, we have had the most unequivocal evidence of injury. We therefore never prescribe it in this disease." p. 367.

Chapter XIX—XX. The author devotes more than 130 pages to the inflammatory affections of the lungs and to consumption.

He has taken advantage of the light thrown upon the subject of diseases of the chest, by the late researches of Laennec, and others; and has collected much valuable information. The chapter is too exclusive to permit even a glance of its contents. We shall merely extract Laennec's account of his mode of administering tartar emitic.

"As soon as I recognise the existence of the pneumonia, if the patient is in a state to bear venesection, I direct from eight to sixteen ounces of blood to be taken from the arm. I very rarely repeat the bleeding, except in patients affected with disease of the heart, or threatened with apoplexy, or some other internal congestion. More than once I have effected very rapid cures of intense peripneumonies without bleeding at all; but, in common, I do not think it right to deprive myself of a means so powerful as venesection, except in chachetic or debilitated subjects. In this respect, M. Rasori does the same. I regard blood-letting as a means of allaying for a time the violence of the inflammatory action, and giving time for the emetic tartar to act. Immediately after bleeding I give one grain of the tartar emetic, dissolved in two ounces and a half of cold, weak infusion of orange leaf, sweetened with half an ounce of sirup of marsh mallows or orange flowers ; and this I repeat every second hour for six times ; after which I leave the patient quiet for seven or eight hours, if the symptoms are not urgent, or if he experiences any inclination to sleep. But if the pneumonia has already made progress, or if the oppression is great, or the head affected, or if both lungs, or one whole lung is attacked, I continue the medicine uninterruptedly, in the same dose and after the same intervals, until there is an amendment, not only in the symptoms, but indicated also in the stethoscopic signs. Sometimes even, particularly when most of the above mentioned unfavorable symptoms are combined, I increase the dose of the tartar emetic to a grain and a half, two grains, or even two grains and a half, without increasing the quantity of the vehicle. Many patients bear the medicine without being either vomited or purged. Others, and indeed the greater number, vomit twice or thrice and have five or six stools the first day ; on the following days they have only slight evacuations, and often indeed have none at all. When once tolerance of the medicine, (to use the expression of Rasori,) is established, it even very frequently happens that the patients are so much constipated as to require clysters to open the body. When the evacuations are continued to the second day, or when there is reason to fear on the first that the medicine will be borne with difficulty, I add to the six doses, to be taken in twenty-four hours, one or two ounces of the sirup of poppies. This combination is in opposition to the theoretical notions of Rasori and Tommasini, but has been proved to me by experience to be very useful. In general the effect of tartar emetic is never more rapid or more efficient than when it gives rise to evacuation ; sometimes, however, its salutary operation is accompanied by a general perspiration. Although copious purging and frequent vomiting are by no means desirable, on account of the debility and hurtful irritation of the intestinal canal which they may occasion, I have obtained remarkable cures in cases in which such evacuations had been very copious. I have met with very few cases of pneumonia where the patient could not bear the emetic tartar ; and the few I have met with occured in my earliest trials ; insomuch that this result now appears to me to be attributable rather to the inexperience and want of confidence of the physician, than to the practice. I now frequently find that the patient who bears only moderately six grains with the sirup of poppies, will bear nine perfectly well the following day. At the end of twenty-four or forty-eight hours at most, frequently after two or three hours, we perceive a marked improvement in all the symptoms." p. 434—435.

In the treatment of phthisis, he recommends bleeding where inflammatory symptoms are present. He condemns all external irritants to the chest, moxo, blisters, setons, issues, tartar antimony , agreeing with Laennec that he has never derived any permanent advantage from them. He laments "the unavailing, and in some instances unfeeling practice, of sending invalids from their kind homes, to die in a strange land, bereft of almost every solace that illness and suffering, so strongly claim." If the following remarks be true, and we have no doubt that they are so generally, how much suffering has been rashly inflicted:

"If the mischief has advanced a little farther, and there are good reasons for believing that tubercles are already formed in the lungs, more especially if a disposition to inflammation of the organs, or to hæmoptysis, has manifested itself; then, change of climate becomes a more doubtful measure: and, unless adopted with judgment, and with some precaution, may accelerate, rather than retard the progress of disease. In cases of this kind, it will be necessary, previously to undertaking the journey, to remove, or at least to moderate, the more evident or important of the functional derangements, to subdue excitement, and diminish plethora. Much evil has arisen from inattention to these precautions. Medical men in general seem hardly sufficiently aware of the great excitement produced in the system by travelling, and of the necessity, therefore, of removing those morbid complications most likely to suffer aggravation from this. If the disease has made still greater progress, and the cough, expectoration, emaciation, hectic fever, and the results of auscultation, leave no doubt of the advanced stage of the tubercles; the mischief to be apprehended from the exposure, the fatigue, the irritation, and excitement of a long journey, is greatly increased; and, under such circumstances, generally speaking, no advantage is to be expected from the change; and very often the fatal termination will be accelerated by it. But should the symptoms just enumerated, from whatever cause, have become much mitigated, and more especially if there is reason to believe, from a careful examination of the chest, that the disease is confined to a small portion of the lungs; theu, a residence in a milder climate affords the best opportunity of aiding the efforts of nature in the work of reparation; and, by contributing to the reestablishment of the general health, will tend to prevent the further formation of tubercles." p. 516—517.

Of all other narcotics, he prefers the preparations of opium for the purpose of relieving the cough.

Here, for the present, we shall take our leave of this work, designing, at a subsequent period, to take a cursory notice of the succeeding chapters. Of the work, as a view of the theory and practice of medicine of the present day, we confess we are rather disappointed. As a work of elementary instruction, calculated to give clear and precise views, not of what learned men have thought, but of what practical men are anxious to know, there is no book which we would place in the hands of the student with more confidence. In comparison with what the author has written heretofore, the present work will appear to disadvantage. The talents and habits of the author we presume are better adapted to mechanical than scientific arrangement. Hence one reason for the superiority of his former works over the present. An additional reason may be found in the fact, that to the subject of the former, he has devoted himself more exclusively, both as a science and as a matter of practice. To the latter, he has devoted himself principally with a view to the acquisition of practical skill.

In the present work, so far as he enters into speculations of this kind, he appears as the advocate of the principles of Broussais, as far as could be expected of a man whose opinions and practice had been formed under the opinions of the old school.

Upon the subject of the universal proscription of active purgatives, we think, even admitting the truth of Broussais' premises, it by no means results that his practice follows as a necessary consequence. If purgatives *are* direct irritants to the mucous membrane of the bowels, do we not frequently prescribe irritants to inflame surfaces with a view to their cure? and independent of any influence of this nature by these medicines, may not the increased secretions which result from their operation, have a powerful influence in controlling or modifying this inflammatory action?

The fact we believe to be, that the mucous surfaces of the primæ viæ in fever, are very generally in a state of irritation, arising from the influence of the general excitement upon organs of great sensibility, possessed of numerous sympathies, and which, in consequence of our artificial modes of living, are debilitated by their constant exposure to the causes of functional and organic irritation. Hence, in fever, local determinations almost universally take place to the mucous surfaces of these organs, as the most sensible, the most exposed, and the most debilitated part of the body; and as a consequence, their functions are impaired, their secretions vitiated, and they are usually in a state of inflammation, or of irritation approaching very nearly to it.

One important indication to be fulfilled by purgatives, we conceive to be the restoration of their secretions. The *modus operandi* of this process, we pretend not to explain. It is sufficient to our purpose, that daily experience proves the *fact.*

That purgatives would be useless or injurious during a state of very high irritation, we would very naturally infer, from what we observe of the effect of every other class of medicines during this condition of the system. Neither diaphoretics, diuretics, blisters, alteratives, nor even those medicines which are in their operation directly debilitating, as arsenic, digitalis, and antimony, can be given with propriety under these circumstances. The secretions are locked up, as a consequence of that condition of the vascular system, which invariably accompanies vascular irritation. Cathartics do indeed admit of a wider range of application, since they are daily used with advantage for the very purpose of preparing the system for the administration of these remedies, but there is doubtless a limit with regard to them also, and he who would avoid the error of adapting his remedies to the name of the disease, must frequently lower the excitement by general or local depletion, before he ventures to give any cathartic, especially drastic ones.

We are very willing to admit, therefore, that the present practice of prescribing this class of remedies universally at the commencement of every disease, requires to be modified; but we are equally certain (although upon this our limited experience entitles our opinion to less weight with the public,) we are equally certain, that in universally avoiding all remedies of this class, they are verging still further upon the opposite extreme.

